# Endoscopic submucosal dissection as an alternative management for huge colon lipoma with acute intussusception

**DOI:** 10.1055/a-2590-7741

**Published:** 2025-05-22

**Authors:** Yu-Zu Lin, Jui-Shen Chang, Wei-Chun Chen, Jeng-Kai Jiang, Shih-Ching Chang, Yuan-Tzu Lan

**Affiliations:** 1 541456 Division of Colon and Rectal Surgery, Department of Surgery, Taipei Veterans General Hospital, Taipei City, Taiwan; 2 46615 Endoscopic Center for Diagnosis and Treatment, Taipei Veterans General Hospital, Taipei City, Taiwan; 3 46615 Faculty of Medicine, School of Medicine, National Yang Ming Chiao Tung University, Taipei City, Taiwan

A 46-year-old patient presented to the emergency department with a 2-day history of abdominal pain. Computed tomography revealed a 7.8 cm hypodense submucosal mass in the descending and sigmoid colon, causing intussusception with fat-stranding, peritoneum-thickening, but no proximal colon distension ( Fig. 1 ). Sigmoidoscopy showed the lesion to be a submucosal tumor with a thick stalk and segmental mucosal congestion around the base, which was compatible with lipoma presenting as acute and reducible intussusception ( Fig. 2 ).

**Fig. 1:**
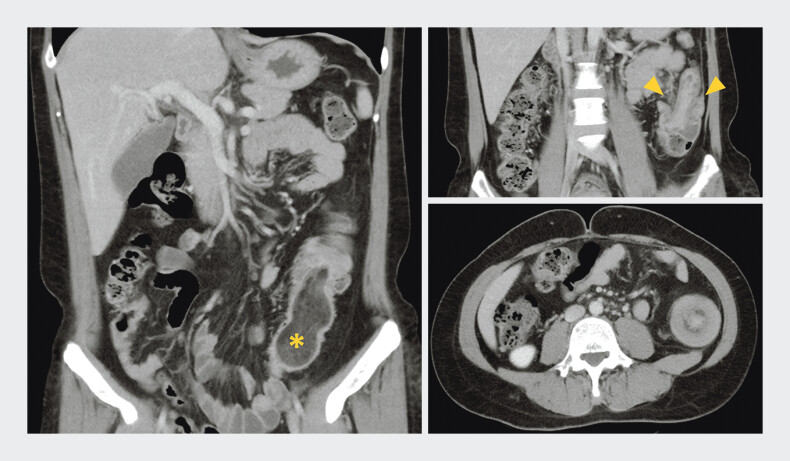
Abdominal computed tomography showing a hypodense submucosal mass (asterisk) in the descending and sigmoid colon with intussusception (arrowhead).

**Fig. 2:**
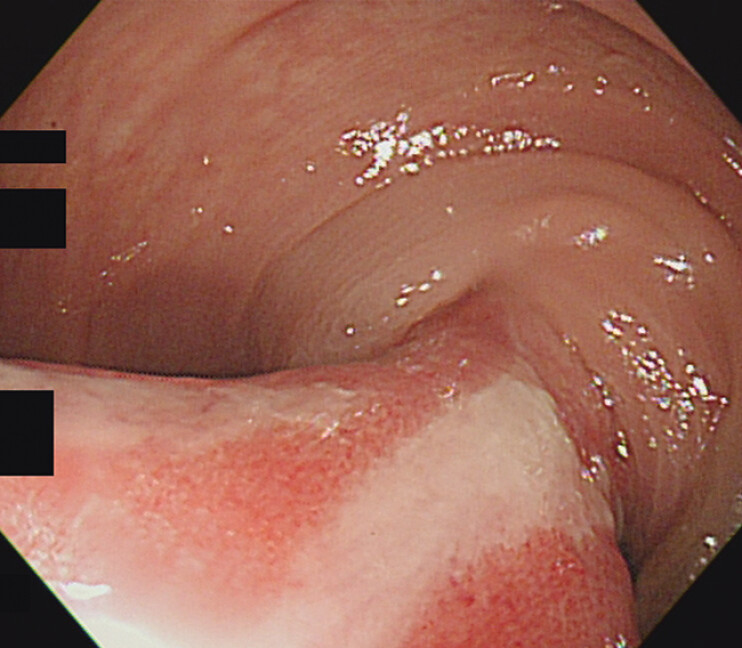
Sigmoidoscopy revealing a huge and elongated submucosal mass.

Endoscopic submucosal dissection (ESD) was performed following standard bowel preparation ( Video 1 ). Due to intussusception, semi-solid stool remained in the colon and interfered with the procedure. The elongated mass rotated by itself, which impaired the orientation and stability of the colonoscope. After submucosal injection, the stalk was incised and dissected with an electrocautery knife uneventfully. The specimen was retrieved with a rigid sigmoidoscopy because it was stuck in the sharp-angled rectosigmoid junction. The procedure took 80 minutes. The specimen measured 8 × 4.5 × 3 cm ( Fig. 3 ). Pathology confirmed lipoma. Follow-up colonoscopy at 3 months showed no recurrence ( Fig. 4 ).

Endoscopic submucosal dissection for colon lipoma with acute intussusception.Video 1

**Fig. 3:**
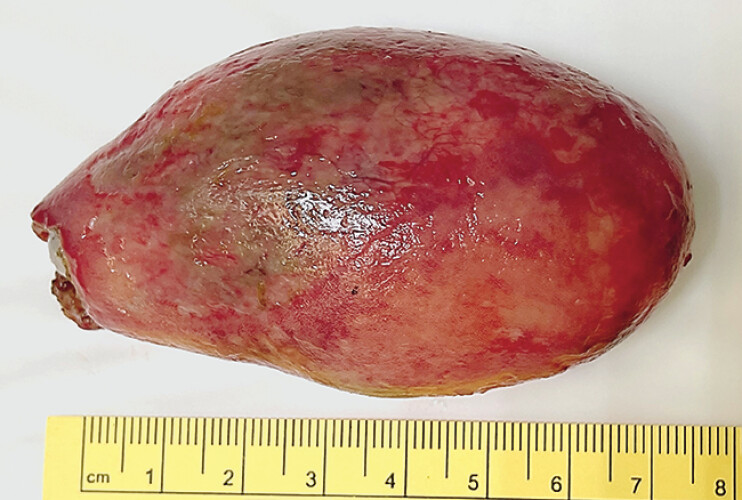
Resected specimen measuring 8 × 4.5 × 3 cm.

**Fig. 4:**
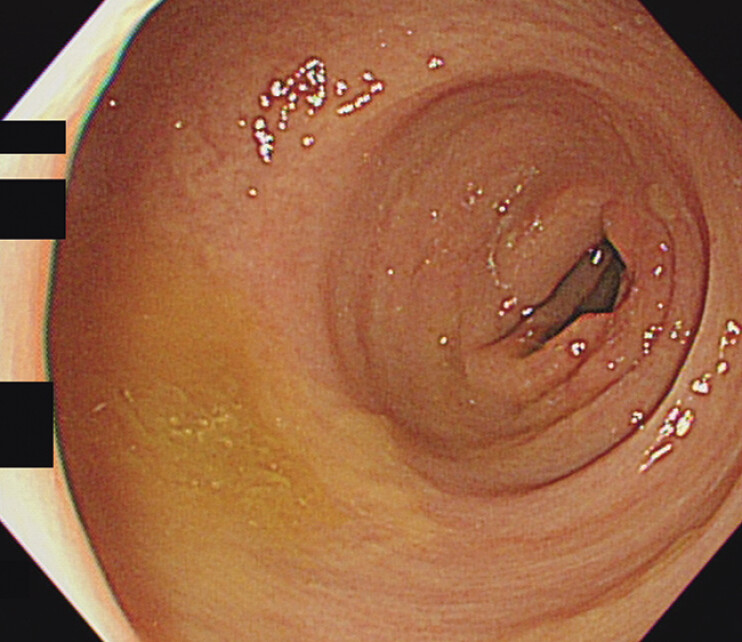
Surveillance colonoscopy 3 months after resection showing scars without signs of recurrence.

Colon lipomas over 20 mm are often symptomatic and require endoscopic or surgical intervention 1 . While colectomy has been the conventional treatment for lipomas complicated with intussusception 2 , recent reports suggest that ESD is a safe and effective alternative, particularly for lipomas with intermittent or chronic intussusception 3 4 . To our knowledge, this is the first description of ESD for colon lipoma with acute intussusception. The acute nature of this case, however, presented unique challenges, including decreased cleansing level, congested mucosa, and instability of the scope manipulation, which were not reported previously.

In conclusion, huge colon lipomas with acute intussusception can be managed by ESD in selected patients when local expertise is available. Collaboration between endoscopists and surgeons is crucial for optimal outcomes.

Endoscopy_UCTN_Code_TTT_1AQ_2AD_3AD

## Notes

Endoscopy E-Videos https://eref.thieme.de/e-videos
*E-Videos* is an open access online section of the journal *Endoscopy* , reporting on interesting cases and new techniques in gastroenterological endoscopy. All papers include a high-quality video and are published with a Creative Commons CC-BY license. Endoscopy E-Videos qualify for HINARI discounts and waivers and eligibility is automatically checked during the submission process. We grant 100% waivers to articles whose corresponding authors are based in Group A countries and 50% waivers to those who are based in Group B countries as classified by Research4Life (see: https://www.research4life.org/access/eligibility/ ). This section has its own submission website at https://mc.manuscriptcentral.com/e-videos .
